# Mortality in long-term care residents: retrospective national cohort study

**DOI:** 10.1136/spcare-2024-005163

**Published:** 2024-10-09

**Authors:** Jane MacRae, Giorgio Ciminata, Claudia Geue, Ellen Lynch, Susan D Shenkin, Terence J Quinn, Jennifer Kirsty Burton

**Affiliations:** 1 Academic Geriatric Medicine, School of Cardiovascular and Metabolic Health, Collge of Medical, Veterinary & Life Sciences, University of Glasgow, Glasgow, UK; 2 Health Economics and Health Technology Assessment, School of Health & Wellbeing, College of Medical, Veterinary & Life Sciences, University of Glasgow, Glasgow, UK; 3 Social Care Analytical Unit, Health and Social Care Analysis, Scottish Government, Edinburgh, UK; 4 Ageing and Health Research Group and Advanced Care Research Centre, Usher Institute, University of Edinburgh, Edinburgh, UK

**Keywords:** Palliative Care, Nursing Home care, Social care

## Abstract

**Objectives:**

Mortality trends among people living in long-term care settings have been poorly understood. Linking data offers the potential to provide real-world, long-term national follow-up. Our aim was to describe patterns and associations with mortality among people moving-in to care homes in Scotland.

**Methods:**

A retrospective cohort study was undertaken using routinely collected national social care data from the Scottish Care Home Census. These data were indexed and linked to national health data and mortality records for individuals moving- in to care homes in Scotland between 1 April 2013 and 31 March 2016. Location of death, underlying causes and time to death are reported. Survival analysis was undertaken using the Gompertz model for human mortality adjusted for key variables.

**Results:**

Of 23 892 individuals moving-in to care homes, 20 250 (84.8%) died by 31 May 2020. Most deaths occurred in the care home setting (77.5%), with a fifth (20.5%) occurring in the hospital. 0.1% died the day they moved-in, 3.2% within a month, 24.2% within a year and 85% by 7 years. Dementia codes account for more than a third of all deaths (35.1%). Median survival time was shorter (701 vs 951 days; 23 vs 32 months) for those moving-in from the hospital, compared with the community. The adjusted HR for moving-in from the hospital was 1.19 (95% CI 1.15 to 1.22).

**Conclusions:**

Mortality is common in Scotland’s care homes but varies in timing. Dementia is the most common cause. Those moving-in from the hospital are more likely to die sooner, and this evidence provides opportunities to improve support for all involved.

**WHAT IS ALREADY KNOWN ON THIS TOPIC**

- Previous studies of mortality from long-term care settings have focused on specific diseases or medications, have compared mortality between residents and matched individuals who live elsewhere in the community or have considered predictors of mortality.

**WHAT THIS STUDY ADDS**

- Mortality is common in Scotland’s care homes and largely occurs within the care home setting.
- There is complexity in supporting the needs of a vulnerable population for whom time within the care home ranges from short weeks to several years.

**HOW THIS STUDY MIGHT AFFECT RESEARCH, PRACTICE OR POLICY**

- The acuity of presentations resulting in hospital deaths indicates significant healthcare needs among this population, which require support to manage equitably.
- Recognising the shorter survival and the most common causes of death among those moving-in from the hospital provides stimulus for targeted support for these individuals, their loved ones and the staff supporting them.

## Background

 There is growing international recognition of the important contribution high-quality long-term care provides for adults with complex needs.^1 2^ Long-term care settings where individuals receive 24-hour residential care and support are heterogeneously named and constructed,^3 4^ with ‘care home’ capturing both those with and without nursing care provision. In both the UK and Scotland, specifically, the role of care homes in caring for those who are dying is recognised as a priority in the face of population ageing and policy supporting death away from hospital settings.^5 6^

There was recognition during the COVID-19 pandemic of the disproportionate impact of the infection on care home mortality rates.^7^ However, as part of wider exposure of the evidence gap in data about people living in care homes,^8^ understanding of usual mortality trends among the care home population was also recognised as missing.^9 10^ Access to contemporaneous data about those dying in care homes has the potential to help support those working in and supporting care homes and to better understand population health and needs.

Harnessing existing routinely collected data for research offers an inclusive mechanism to study the population living in care homes at scale without additional burden to staff or residents. However, such research relies on representative data, which reliably identifies the whole population.^11^ The Scottish Care Home Census (SCHC) is a national data resource of information about care home services and residents that has been underused for research.^12^ We have previously demonstrated the validity of linking these data to explore hypotheses around pathways into care homes and health economics.^13 14^ Here we focus on understanding care home mortality at scale. Our aim was to describe patterns and associations with mortality among people moving-in to care homes in Scotland.

## Methods

### Study design

We performed a retrospective observational cohort study of adults moving-in to care homes in Scotland in the financial years 2013/2014–2015/2016. National mortality data were linked to our cohort of care home residents, with follow-up to 31 May 2020.

### Setting and context

The term ‘care home’ is used in this paper inclusively to describe long-term care facilities that provided 24-hour care and support to adults in Scotland with complex needs. Around three-quarters of registered services and >90% of beds are provided for older people.^15^ Other services are focused on supporting adults with addictions, mental health problems, learning disability and physical and sensory impairments.^15^ Care homes in Scotland provide temporary, short- and long-stay provision (typically defined at 6 weeks or longer). This analysis uses data on those classified as long-stay residents.

### Indexing and linkage methodology

Individuals were included in the study if they had an SCHC record indicating they moved-in to a care home in Scotland from a hospital or from the community between 1 April 2013 and 31 March 2016. This was determined through linkage between the SCHC data and other national data sources (full cohort definition published previously).^13^ Those moving-in from another care home were not included, so valid comparisons of survival after moving-in to the home could be made.

The linkage between datasets requires the use of the Scottish national person-level identifier variable, the Community Health Index (CHI) number. An indexing process was undertaken separately from the research team to add the CHI number to SCHC data to facilitate linkage to other national data (full methods are reported elsewhere).^12^ Records in the SCHC that could not be indexed to CHI were excluded (3.9% of all records available for analysis).^13^ Deidentified data extracts were made available in the National Safe Haven for remote researcher access, linkage and analysis.

## Data sources

*CHI register*: nationally held population spine, which includes all individuals in Scotland registered with a general practitioner, using their 10-digit unique identifier, the CHI number.^16^

*National Records of Scotland (NRS) mortality records*: national mortality records of deaths registered in Scotland from 1 April 2013 to 31 May 2020.

*SCHC*: annual national data collection from care homes in Scotland about the activity of the care home over the preceding financial year (1 April–31 March). SCHC data for financial years 2013/2014, 2014/2015 and 2015/2016 were used.

*Scottish morbidity records (SMR) 01, 04 and 50*: general acute inpatients and day case records; mental health inpatients and day case records and geriatric long-stay records from Scottish hospitals; extract of admissions from 1 April 2010 to 31 May 2016.

### Variables for analysis, by data source

*CHI register*: adjusted date of birth and sex.

*SCHC*: ethnicity, funding, dependency (receiving nursing care within the care home or not), care home service subtype, financial year of SCHC, date moving into care home.

*NRS*: date of death, location of death (using location codes—hospital—C and H codes. Care home—J, K, R and V. Non-institution—N. Other institution—P, S and T).^17^ Underlying cause of death. We included both individual International Classification of Diseases 10th edition (ICD-10) codes but also grouped codes into broader causes of deaths to facilitate meaningful clinical comparisons. Code groupings are presented in online supplemental table 1.

*Derived*: frailty status (based on Hospital Frailty Risk Score)^18^; comorbidity (Charlson Index)^19 20^; moving-in from hospital or community (allocation method described in full previously)^13^; time to death after moving-in to care home (using NRS date of death and SCHC date of care home admission).

Early mortality was defined as within the first 180 days (6 months) after moving-in to the care home. Causes of death in this early period were compared with deaths occurring later to explore if there were observable patterns or important differences.

### Statistical methods

The Kaplan-Meier estimator was used to generate non-parametric survival functions. A parametric model using the Gompertz distribution, reflecting human mortality, was developed to estimate mortality risk, adjusting for age, sex and frailty status. We compared those moving-in to the care home from the hospital with those moving-in from the community and reported their HRs and median survival time. Analyses were conducted in Stata (V.16.1, StataCorp LLC, College Station, Texas, USA).

### Sensitivity analysis

Sensitivity analysis was undertaken, removing those aged <60 years at the time of moving-in to the care home from the survival analysis. This was to examine the impact of younger people on the mortality analysis for the whole study cohort.

### Permissions and governance

Ethical approval was obtained from the South Central-Hampshire B Research Ethics Committee (16/SC/0242). Permission for linking national data was granted by the Public Benefit and Privacy Panel for Scotland (1516-0438) and the Scottish Government Social Care Analysis Division.

All outputs from the National Safe Haven were subject to information governance and statistical disclosure control by the electronic data research and innovation service.

### Public and stakeholder involvement

The terminology ‘moving in’ is purposively used throughout the manuscript to address feedback from public and care home stakeholder contributors that individuals are not ‘admitted’ to their homes. Thus, clinical terminology around transitions of care is avoided. Engagement visits to care homes identified the utility of the data generated in this study and its relevance to everyday practice from the perspective of residents, their loved ones and the professionals supporting them.

## Results

### Summary of cohort characteristics

Our study cohort includes 23 892 individuals moving into care homes between 1 April 2013 and 31 May 2016, of whom 20 250 (84.8%) died by 31 May 2020.

Those who died with those who survived are compared in table 1. Survivors were more likely to be younger, less dependent and at lower risk of frailty. Most deaths occurred in older people’s care home services (87.2% survived to the end of follow-up). A lower proportion of those with mental health problems survived (67.7%) compared with care homes for those with physical and sensory impairment (76.3%) or learning disabilities (82.7%), although the total numbers in these groups were small. A greater proportion of those moving-in to the care home from the hospital died, compared with those moving in from the community (87.8 vs 80.8%, a difference of 7 percentage points; 95% CI 6.1% to 7.9%).

**Table 1:** Descriptive analysis of cohort comparing those who died with those who survived

	Died N=20 250		Survived N=3642	
	Number	% by group	Number	% by group
Age band moving into care home				
<60 years	342	29.3	826	70.7
60–79 years	704	63.9	397	36.1
70–79 years	3453	80.8	821	19.2
80–89 years	9841	88.8	1246	11.2
90–99 years	5689	94.4	340	5.6
>100 years	221	94.8	12	5.2
Sex				
Female	7051	85.0	1248	15.0
Male	13 199	84.6	2394	15.4
Ethnicity				
White ethnic group	19 545	85.0	3458	15.0
Other ethnic group	327	76.6	100	23.4
Ethnic group not reported	378	81.8	84	18.2
Funding				
Mainly local authority	13 037	83.2	2633	16.8
Mainly National Health Service	542	88.6	70	11.4
Mainly private	6653	87.7	935	12.3
Missing funding variable	18	81.8	4	18.2
Dependency				
Receiving nursing care in care home	13 632	87.8	1900	12.2
Not receiving nursing care in care home	6606	79.2	1736	20.8
Missing nursing care variable	12	66.7	6	33.3
Hospital Frailty Risk Score				
Low risk (<5)	4613	78.8	1243	21.2
Intermediate risk (5-15)	8458	87.5	1205	12.5
High risk (≥15)	5835	91.0	577	9.0
Incalculable*	1344	68.5	617	31.5
Charlson Index				
0 comorbidities	5320	78.7	1441	21.3
1 comorbidity	6285	86.3	994	13.7
≥1 comorbidities	7301	92.5	590	7.5
Incalculable*	1344	68.5	617	31.5
Care home service subtype				
Learning disabilities	42	17.3	201	82.7
Mental health problems	84	32.3	176	67.7
Older people	20 014	87.2	2940	12.8
Physical and sensory impairment	76	23.8	244	76.3
Other†	34	29.6	81	70.4
Financial year moving-in				
2013/2014	7343	88.7	940	11.3
2014/2015	6830	84.5	1255	15.5
2015/2016	6077	80.8	1447	19.2
Moving-in to care home				
From hospital	11 906	87.8	1658	12.2
From community	8344	80.8	1984	19.2

* Unable to calculate as no hospital admissions data in 3 years prior to moving-in to the care home to base Hospital Frailty Risk Score and Charlson calculations.

† Other care home service subtype includes alcohol and drug misuse; blood-borne virus and respite and short breaks.

The mean age at death in the cohort was 87 years (SD 8.77; range 19–109 years). The mean age at death was 86 years (SD 8.67) in those moving-in to the care home from hospital compared with 87 years (SD 9.03) in those moving in from the community.

### Location of death

Most deaths occurred in the care home setting (77.5%) with a fifth occurring in hospital settings (20.5%). The remaining deaths occurred in a non-institutional setting (ie, a private address) (1.2%) or another institutional setting (0.8%). The distribution of location of death was comparable irrespective of where an individual moved-in to the care home from originally (table 2).

**Table 2:** Location of death

Location of death	All deaths included in the cohort N (%)	Deaths among those moving-in from hospital N (%)	Deaths among those moving-in from the community N (%)
Hospital setting	4151 (20.5)	2441 (20.5)	1710 (20.5)
Care home setting	15 687 (77.5)	9258 (77.8)	6429 (77.0)
Non-institutional setting (ie, private address)	250 (1.2)	117 (1.0)	133 (1.6)
Other institutional setting	162 (0.8)	90 (0.8)	72 (0.9)
Total deaths by group	20 250	11 906	8344

### Causes of death

Dementia codes (inclusive of Alzheimer’s disease, vascular, unspecified and mixed dementia) are the underlying cause of more than a third of all deaths (35.1%) and are the top four causes of all deaths. This is followed by acute myocardial infarction (3.8%), stroke (3.7%), cerebrovascular disease (3.6%) and chronic ischaemic heart disease (2.5%). Falls account for 1.7% of deaths and frailty for 1.2%. The top 30 ICD-10 codes assigned as the underlying cause of death are reported in table 3 comparing those in the cohort as a whole and those whose deaths occurred in hospitals. Dementia codes cumulatively account for 20.5% of hospital deaths; however, other common causes in this group include myocardial infarction, acute infections, falls and stroke. The range of underlying causes of death is broadly similar among those moving-in from the hospital and those moving-in from the community, with differences in their frequency (online supplemental table 2).

**Table 3:** Comparing top 30 underlying causes of death in the whole cohort with those who died in hospital

	Deaths in whole cohort (n=20 250)		Deaths in those who died in a hospital setting (n=4151)	
	ICD-10 code—condition	Number	ICD-10 code—condition	Number
**1**	**F03**—unspecified dementia	2365	**F03**—unspecified dementia	351
**2**	**F01.9**—vascular dementia	2203	**F01.9**—vascular dementia	276
**3**	**G30.9**—Alzheimer’s disease	1708	**I21.9**—acute myocardial infarction	187
**4**	**G30.1**—Alzheimer’s disease, late onset	837	**J18.9**—pneumonia	176
**5**	**I21.9**—acute myocardial infarction	765	**G30.9**—Alzheimer disease	167
**6**	**I64**—stroke, not specified as haemorrhage or infarction	761	**J44.0**—chronic obstructive pulmonary disease with acute lower respiratory infection	152
**7**	**I69.8**—sequalae of cerebrovascular disease	725	**I64**—stroke, not specified as haemorrhage or infarction	148
**8**	**I25.9**—chronic ischaemic heart disease	510	**J69.0**—pneumonitis due to food or vomit	147
**9**	**J44.0**—chronic obstructive pulmonary disease with acute lower respiratory infection	475	**W19**—unspecified fall	147
**10**	**G20**—Parkinson’s disease	407	**I25.9**—chronic ischaemic heart disease	102
**11**	**W19**—unspecified fall	348	**I63.9**—cerebral infarction	93
**12**	**J18.0**—bronchopneumonia	345	**I69.8**—sequalae of cerebrovascular disease	79
**13**	**J18.9**—pneumonia	327	**N39.0**—Urinary tract infection	74
**14**	**C34.9**—malignant neoplasm—bronchus or lung	279	**G20**—Parkinson’s disease	71
**15**	**J98.8**—other specified respiratory disorders	258	**I48**—paroxysmal atrial fibrillation	62
**16**	**R54**—age-related physical debility (frailty)	252	**A41.9**—sepsis	59
**17**	**J22**—unspecified acute lower respiratory tract infection	242	**G30.1**—Alzheimer’s disease, late onset	59
**18**	**N39.0**—urinary tract infection	224	**I61.9**—intracerebral haemorrhage	50
**19**	**J69.0**—pneumonitis due to food or vomit	221	**J18.1**—lobar pneumonia	50
**20**	**J44.9**—chronic obstructive pulmonary disease	219	**I69.4**—sequalae of stroke, not specified as haemorrhage or infarction	46
**21**	**I69.4**—sequalae of stroke, not specified as haemorrhage or infarction	200	**J44.9**—chronic obstructive pulmonary disease	45
**22**	**C50.9**—malignant neoplasm breast	194	**J18.0**—bronchopneumonia	40
**23**	**I48**—paroxysmal atrial fibrillation	191	**J98.8**—other specified respiratory disorders	35
**24**	**I67.9**—cerebrovascular disease	190	**K92.2**—gastrointestinal haemorrhage	35
**25**	**I63.9**—cerebral infarction	184	**C34.9**—malignant neoplasm—bronchus or lung	34
**26**	**C61**—malignant neoplasm of prostate	181	**I73.9**—peripheral vascular disease	34
**27**	**G31.8**—other specified degenerative diseases of nervous system	132	**J22**—unspecified acute lower respiratory tract infection	34
**28**	**I73.9**—peripheral vascular disease	118	**J44.1**—chronic obstructive pulmonary disease with acute exacerbation	32
**29**	**E11.9**—type 2 diabetes without complications	112	**N17.9**—acute renal failure	31
**30**	**K92.2**—gastrointestinal haemorrhage	102	**K31.8**—gastrectasis	30

ICD-10, International Classification of Diseases, 10th edition.

### Time to death

For the 20 250 people who died, time to death ranged from 0 days to 2575 days (7.05 years). The median time to death was 644 days (IQR 892) (1.7 years). The distribution of time to death was further described by clinically meaningful periods (online supplemental table 3). This identified that 0.1% died on the day of moving in, 0.6% within a week and 3.2% within a month. By 6 months, 14.5% had died and at a year, this was 24.2% of the cohort. A third died within 18 months of moving-in and more than half died within 3 years.

**Figure 1:**
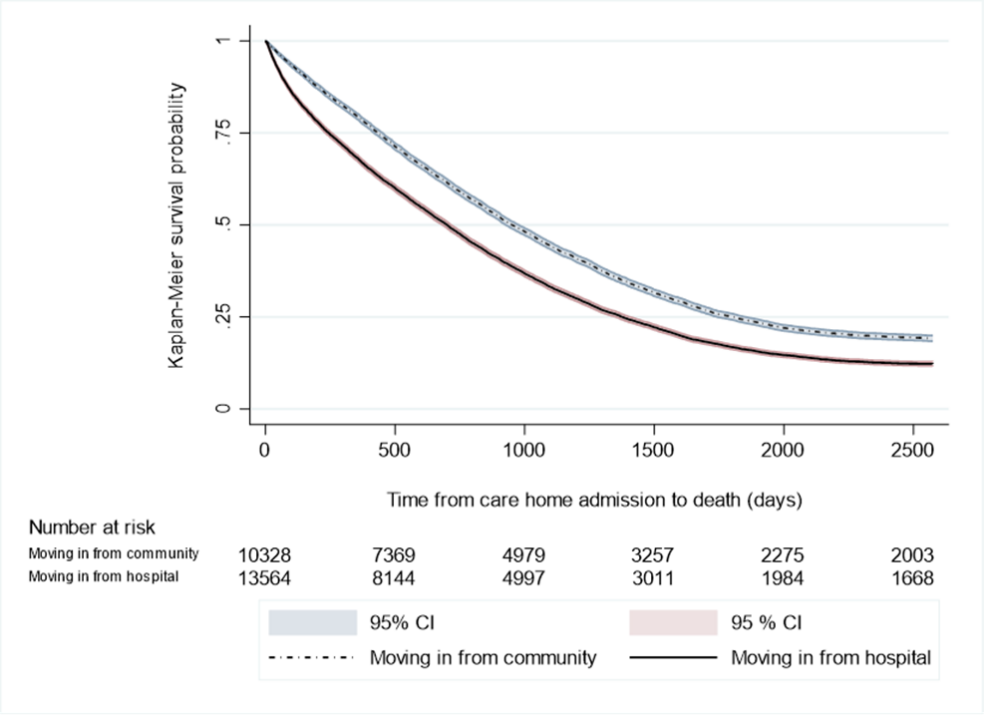
Survival analysis of time from moving-in to the care home to death, comparing those moving-in from the community to those moving-in from the hospital.

Kaplan-Meier survival curves were generated comparing the survival time between moving-in to the care home and death based on where individuals moved-in from. Those moving-in from the hospital have consistently shorter survival times than those moving-in from the community (figure 1). The median survival time is shorter at 701 days (95% CI 683 to 714 days) for those moving-in from the hospital, compared with 951 days (95% CI 928 to 978 days) for those moving-in from the community (23 vs 32 months). The survival model, adjusted for age, sex and frailty, is summarised in table 4. The adjusted HR for death for those moving-in from the hospital is 1.19 (95% CI 1.15 to 1.22). Sensitivity analysis removing those of younger age provided a consistent direction and comparable magnitude of effect (table 4, online supplemental figure 1).

**Table 4:** Summary of survival model data

	Main analysis	Sensitivity analysis*
Total number of events		
Moving-in from community	8344	8189
Moving-in from hospital	11 906	11 719
Event rate (per 100 person years)		
Moving-in from community	0.07	0.08
Moving-in from hospital	0.09	0.1
Median survival time in days (95% CI)		
Moving-in from community	951 (928 to 978)	888 (864 to 907)
Moving-in from hospital	701 (683 to 714)	676 (658 to 694)
Risk of death by group—HR (95% CI)		
Moving-in from community	Reference group	
Moving-in from hospital	1.19 (1.15 to 1.22)	1.18 (1.15 to 1.22)
Risk of death by age—HR (95% CI)		
<60 years	Reference group	–
60–69 years	2.86 (2.51 to 3.25)	Reference group
70–79 years	4.77 (4.26 to 5.33)	1.67 (1.54 to 1.81)
80–89 years	6.50 (5.82 to 7.25)	2.29 (2.12 to 2.47)
90–99 years	8.87 (7.94 to 9.92)	3.13 (2.89 to 3.39)
>100 years	9.99 (8.43 to 11.85)	3.52 (3.02 to 4.10)
Risk of death by sex—HR (95% CI)		
Female	Reference group	
Male	1.41 (1.37 to 1.46)	1.43 (1.38 to 1.47)
Risk of death by frailty—HR (95% CI)		
Low risk	Reference group	
Intermediate risk	1.18 (1.14 to 1.22)	1.16 (1.12 to 1.20)
High risk	1.27 (1.22 to 1.32)	1.25 (1.20 to 1.30)

* Removing those aged under 60 years from survival model.

### Comparing early and later mortality

We grouped ICD-10 codes to facilitate meaningful clinical comparisons (online supplemental table 1); the 3952 deaths occurring ‘early’ were compared with the 16 298 later deaths (table 5). Twelve code groups accounted for 94.1%–95.2% of all underlying causes of death codes used in the cohort. Although dementia is the most common cause of death in both groups, it accounted for 19.2% of early deaths versus 40.2% of later deaths. Cancer, cardiovascular and respiratory causes were commoner as an underlying cause of death in early deaths (cancer 17.6% vs 6.7% of later deaths; cardiovascular 15.9% vs 10.3%; respiratory 15.6% vs 11.6%). Stroke and cerebrovascular disease, neurodegenerative diseases (excluding dementia) and falls were similarly distributed between early and later deaths.

**Table 5:** Comparing underlying causes of death between those experiencing early mortality (within 180 days of moving-in) to later mortality

	ICD-10 codes	Classification	Early (within 180 days)		Later (thereafter)	
			3952 Deaths		16 298 Deaths	
			N	%	N	%
1	F00–03, G30 and G31	Dementia	758	19.2	6550	40.2
2	C00–97	Cancer	694	17.6	1092	6.7
3	I00–52	Cardiovascular	629	15.9	1681	10.3
4	J00–99	Respiratory	616	15.6	1887	11.6
5	G45, I60–69	Stroke and cerebrovascular disease	473	12.0	1887	11.6
6	N00–99	Genitourinary	132	3.3	346	2.1
7	A09, K00–93	Gastrointestinal	124	3.0	521	3.2
8	A81.0, F02.1, F02.2, G10–11, FG20–26, G35–37, G70–73, G90–95	Neurodegenerative diseases (excluding dementia)	105	2.7	483	3.0
9	W00–19, R29.6	Falls	70	1.8	278	1.7
10	E10–14	Diabetes	82	2.1	276	1.7
11	I70–79	Other arterial and vascular causes	48	1.2	119	0.7
12	R54	Frailty	32	0.8	220	1.3
**Total deaths covered by top 12 codes**			**3763**	**95.2**	**15 340**	**94.1**

ICD-10, International Classification of Diseases, 10th edition.

## Discussion

### Findings in context

National data on mortality trends for the population living in Scotland’s care homes show that mortality is common and largely occurs within the care home. Dementia is the most common cause, accounting for more than a third of all deaths, with cardiovascular and cerebrovascular diseases important. Time to death shows significant variation, from 0.1% dying the day they move-in, 3.2% within a month and 24.2% within a year. Survival is significantly shorter in those moving in from the hospital at 23 versus 32 months for those moving-in from the community. Cancer is a significant underlying cause of early mortality (within 6 months). Within Scotland as a whole, ischaemic heart disease, cerebrovascular disease and cancer are leading causes of mortality, although dementia is recognised to be growing in importance with population ageing.^6^

Our findings are consistent with other work in this area. Prior research in a region of Italy identified an increased risk of mortality in the initial months after residents moved-in, associated with recent hospital admission.^21^ Similarly, European data on length of stay in long-term care settings showed that moving-in from the hospital and having a diagnosis of cancer were associated with a shorter length of stay within the long-term care facility.^22^ While no other studies have specifically compared survival based on where individuals moved-in from, the association with prior hospitalisations before moving-in to the care home as a marker of increased mortality risk has been identified elsewhere.^23^ Although in our cohort most people died in the care home, the fifth who died in the hospital represent an important group, adding to the evidence around the complexity of needs and the need for tailored support to respond to acute deterioration in residents’ health,^24^ noting the greater acuity in causes within this subgroup. Recent Canadian data have suggested variation in the location of death in this population based on the urban/rural status of the home,^25^ another potential hypothesis to explore further. Others have encountered challenges in trying to predict mortality risk in this population.^26^ While not our focus, the differences observed within the cohort do provoke reflection on the information available to support people moving-in to care homes and their families in terms of anticipatory care planning.

### Strengths and limitations

We have demonstrated the feasibility of constructing a national retrospective cohort study linking routinely collected social care data, collected by care home staff for the SCHC, to health and mortality data. The linkages undertaken to enhance the information available, compared with the primary data source alone, include significantly extending follow-up. The cohort is inclusive of individuals resident in care homes and is not reliant on proxy methods of identification, such as address matching or general practitioner (GP) registration, both of which have limitations.^27^ The cohort is also inclusive of people who may lack the capacity to consent to provide their information, but it enables them to contribute to a comprehensive knowledge of the sector within a strict ethical/governance framework.

Important limitations to acknowledge are that reporting individual-resident data to the SCHC is not mandatory, thus not all individuals will be captured. For the financial years of our dataset, 74%–81% of open care homes contributed data; this underestimates completeness as it includes homes that do not have long-stay residents.^28^ As the focus of the collection is those whose stay was planned as a long stay (6 weeks or longer),^12^ our sample may under-represent those whose stay was very short, although the findings include some people who died on the day they moved-in. Routine data do not include information on the severity of conditions (such as dementia) to allow greater nuanced analysis. Similarly, achieving meaningful groupings of ICD-10 codes for mortality can be difficult without losing too much detail. We do not have data on the palliative care support received by our cohort; primary care data are not nationally available in Scotland to enable linkage to palliative care registers. Similarly, we did not link our data to community pharmacy dispensing data to explore access to medications at the end-of-life/anticipatory prescribing. This could form an area for further research.

### Implications for policy, practice and research

These findings provide evidence that caring for the dying is very much an everyday part of care home life. It is therefore imperative that care home staff are provided with support to equip them to undertake this aspect of their role, including support in managing the impact of loss and bereavement.^29^

The dominance of dementia as a cause of death in this population also provokes further reflection, given the lack of access to palliative care support for this population, despite significant symptom burden.^30^ This must be a priority for future research and practice development to ensure equitable access.

The clear differences in the experiences of those moving-in from the hospital compared with those moving-in from the community provide further evidence of the need to consider the different needs of these two groups in terms of planning interventions and support around long-term care transitions and utilisation of services.

This work shows the potential positive contribution that high-quality, inclusive data can have to inform the system-level response to support people living in care homes. This includes practice development, education and training opportunities for home care staff and the healthcare team supporting this population in primary and community care, including GPs and district nurses. It can support resource allocation to take account of the complexity of needs, ensuring services can respond. It can also help inform service planning from a population health perspective, ensuring places are funded to support the delivery of high-quality end-of-life care for people living and dying in care homes.

## Conclusions

Mortality among people living in long-term care settings is common but varied in time and causes and requires greater understanding. Having specific data and focussing on causes of death, location and time to death can assist in the planning of services, resources and support for professionals working in and with the long-term care sector. Recognising the shorter survival among those moving-in from the hospital provides stimulus for targeted support for these individuals, their loved ones and the staff supporting them.

## Supplementary material

10.1136/spcare-2024-005163 online supplemental file 1

## Data Availability

Data may be obtained from a third party and are not publicly available. The datasets used in this study are collected/held/controlled by Public Health Scotland, National Records of Scotland, the Care Inspectorate and Scottish Government. Data access can be enabled by application to the Public Benefit and Privacy Panel for Health and Social Care and eDRIS team (Public Health Scotland).
