# Supplementary material for: Mortality in long-term care residents: retrospective national cohort study
Source: BMJ Support Palliat Care. 2024 Oct 9;16(1):e005163. doi: 10.1136/spcare-2024-005163 (PMC12772553; doi:10.1136/spcare-2024-005163)
Supplement: online supplemental file 1 [file spcare-16-1-s001.pdf]

Supplementary Materials

	Page number
**Supplementary Table 1: ICD-10 Mortality Code Groupings**	**2**
**Supplementary Table 2: Comparing underlying causes of death among those moving-in from hospital and those moving-in from the community**	**3**
**Supplementary Table 3: Cumulative time to death after moving-in to care home**	**4**
**Supplementary Figure 1: Sensitivity analysis survival analysis of time from moving-in to the care home to death comparing those moving-in from the community to those moving-in from hospital excluding those aged <60 years when moving-in**	**5**

Supplementary Table 1: ICD-10 Mortality Code Groupings

- Cancer (C00-97)
- Cardiovascular (I00-52)
- Congenital disease including Downs Syndrome (Q00-99)
- COVID-19 (U071, U072)
- Dementia (F00, F01, F02, F03, G30, G31)
- Delirium (F05)
- Depression (F32, F33)
- Diabetes (E10-14)
- Digestive system (K00-93)
- Epilepsy (G40, G41)
- Falls (W00-19, R29.6)
- Frailty (Age-related physical debility) (R54)
- Genitourinary (N00-99)
- Haematological (D45, D46, D50-89)
- Mental and behavioural disorders due to use of alcohol (F10)
- Mental and behavioural disorders due to use of psychoactive substances (F11-19)
- Musculoskeletal and connective tissue (M00-99)
- Neurodegenerative disease including Parkinson's Disease *(excluding dementia)* (A81.0, F02.1, F02.2, G10-14, G20-26, G35-37, G70-73, G90-95)
- Other arterial and vascular causes including dissection and peripheral vascular disease (I70-79)
- Respiratory infection (J00-06; J09-18; J20-22; J44.0; J69)
- Respiratory (non-infective) (J30-99; excluding J44.0 & J69)
- Schizophrenia (F20) & Schizoaffective disorders (F25)
- Stroke & Cerebrovascular disease (G45, I60, I61-69)

AND Other (all other ICD-10 codes; excluding those used above)

Supplementary Table 2: Comparing underlying causes of death among those moving-in from hospital and those moving-in from the community

	**Deaths in those moving-in from hospital**		**Deaths in those moving-in from the community**	
	ICD-10 code – Condition	*Number*	*ICD-10 code – Condition*	*Number*
**1**	**F03** – Unspecified dementia	1358	**F03** – Unspecified dementia	1007
**2**	**F01.9** – Vascular dementia	1305	**F01.9** – Vascular dementia	898
**3**	**G30.9** – Alzheimer disease	825	**G30.9** – Alzheimer disease	883
**4**	**I64** – Stroke, not specified as haemorrhage or infarction	450	**G30.1** – Alzheimer disease, late-onset	456
**5**	**I69.8** – Sequalae of cerebrovascular disease	449	**I21.9** – Acute myocardial infarction	320
**6**	**I21.9** – Acute myocardial infarction	445	**I64** – Stroke, not specified as haemorrhage or infarction	311
**7**	**G30.1** – Alzheimer disease, late-onset	381	**I69.8** – Sequalae of cerebrovascular disease	276
**8**	**J44.0** – Chronic obstructive pulmonary disease with acute lower respiratory infection	321	**I25.9** – Chronic ischaemic heart disease	206
**9**	**I25.9** – Chronic ischaemic heart disease	304	**W19** – Unspecified fall	167
**10**	**G20** – Parkinson disease	259	**J44.0** – Chronic obstructive pulmonary disease with acute lower respiratory infection	154
**11**	**J18.0** – Bronchopneumonia	223	**G20** – Parkinson disease	148
**12**	**J18.9** – Pneumonia	207	**J18.0** – Bronchopneumonia	122
**13**	**C34.9** – Malignant neoplasm – bronchus or lung	195	**J18.9** – Pneumonia	120
**14**	**W19** – Unspecified fall	181	**R54** – Age-related physical debility (Frailty)	119
**15**	**J98.8** – Other specified respiratory disorders	157	**J98.8** – Other specified respiratory disorders	101
**16**	**I69.4** – Sequalae of stroke, not specified as haemorrhage or infarction	146	**J22** – Unspecified acute lower respiratory tract infection	99
**17**	**J22** – Unspecified acute lower respiratory tract infection	143	**J69.0** – Pneumonitis due to food or vomit	98
**18**	**J44.9** – Chronic obstructive pulmonary disease	141	**N39.0** – Urinary tract infection	91
**19**	**N39.0** – Urinary tract infection	133	**C34.9** – Malignant neoplasm – bronchus or lung	84
**20**	**R54** – Age-related physical debility (Frailty)	133	**J44.9** – Chronic obstructive pulmonary disease	78
**21**	**C61** – Malignant neoplasm of prostate	130	**I63.9** – Cerebral infarction	73
**22**	**I67.9** – Cerebrovascular disease	129	**C50.9** – Malignant neoplasm breast	70
**23**	**C50.9** – Malignant neoplasm breast	124	**I48** – Paroxysmal atrial fibrillation	68
**24**	**I48** – Paroxysmal atrial fibrillation	123	**G31.8** – Other specified degenerative diseases of nervous system	62
**25**	**J69.0** – Pneumonitis due to food or vomit	123	**I67.9** – Cerebrovascular disease	61
**26**	**I63.9** – Cerebral infarction	111	**I69.4** – Sequalae of stroke, not specified as haemorrhage or infarction	54
**27**	**I73.9** – Peripheral vascular disease	74	**C61** – Malignant neoplasm of prostate	51
**28**	**E11.9** – Type two diabetes without complications	71	**I10** – Essential hypertension	44
**29**	**G31.8** – Other specified degenerative diseases of nervous system	70	**I489** – Atrial fibrillation and atrial flutter	44
**30**	**K92.2** – Gastrointestinal haemorrhage	64	**I73.9** – Peripheral vascular disease	44

Supplementary Table 3: Cumulative time to death after moving-in to care home

Time period	Number of individuals who have died (% of cohort)
**Day of moving-in**	20 (0.1)
**Within a week** (0 to 7 days)	154 (0.6)
**Within a month** (0 to 30 days)	869 (3.2)
**Within three months** (0 to 90 days)	2353 (8.6)
**Within six months** (0 to 180 days)	3952 (14.5)
**Within nine months** (0 to 270 days)	5290 (19.4)
**Within one year** (0 to 365 days)	6603 (24.2)
**Within eighteen months** (0 to 548 days)	8991 (33.0)
**Within two years** (0 to 730 days)	11133 (40.9)
**Within three years** (0 to 1095 days)	14781 (54.3)
**Within four years** (0 to 1460 days)	17389 (63.8)
**Within five years** (0 to 1825 days)	19116 (70.2)

Supplementary Figure 1: Sensitivity analysis survival analysis of time from moving-in to the care home to death comparing those moving-in from the community to those moving-in from hospital excluding those aged <60 years when moving-in

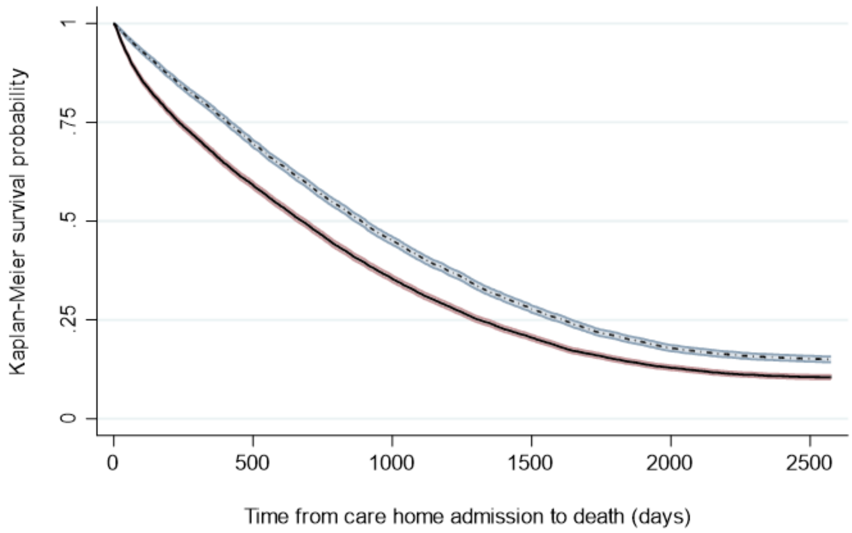

Number at risk

Moving in from community	9635	6707	4341	2674	1723	1464
Moving in from hospital	13089	7738	4638	2686	1684	1380

95% CI — Moving in from community; 95% CI — Moving in from hospital
